# Clinical experience with distal transradial access for endovascular treatment of various noncoronary interventions in a multicenter study

**DOI:** 10.1371/journal.pone.0237798

**Published:** 2020-08-21

**Authors:** Sung Eun Park, Soo Buem Cho, Hye Jin Baek, Jin Il Moon, Kyeong Hwa Ryu, Ji Young Ha, Sangmin Lee, Jungho Won, Jong-Hwa Ahn, Ran Kim, Sun Young Choi

**Affiliations:** 1 Department of Radiology, Gyeongsang National University School of Medicine and Gyeongsang National University Changwon Hospital, Changwon, Republic of Korea; 2 Department of Radiology, College of Medicine, Ewha Womans University, Seoul, Republic of Korea; 3 Department of Radiology, Gyeongsang National University School of Medicine and Gyeongsang National University Hospital, Jinju, Republic of Korea; 4 Department of Internal Medicine, Gyeongsang National University School of Medicine and Gyeongsang National University Changwon Hospital, Changwon, Republic of Korea; Baylor Scott and White, Texas A&M College of Medicine, UNITED STATES

## Abstract

**Background:**

Transradial access is a well-known alternative to conventional transfemoral access for interventional procedures. Recently, transradial access through the “snuffbox”, which lies in the radial dorsal aspect of the hand, has been introduced as a new technique with positional versatility. In this study, we aimed to evaluate the clinical feasibility and safety of distal transradial access for interventional procedures in a retrospective, multicenter study.

**Material & methods:**

Distal transradial access was attempted in 46 patients (36 men and 10 women; mean age, 64 years) who underwent 47 consecutive procedures from January 2018 to December 2019. Procedures included chemoembolization (19/47, 40.4%), bronchial artery embolization (7/47, 14.9%), renal intervention (3/47, 6.4%), arteriovenous fistula angioplasty (7/47, 14.9%), subclavian artery stenting (5/47, 10.6%), other embolization (5/47, 10.6%), and uterine artery embolization (1/47, 2.1%). We recorded the success rate of the procedures, complications, and postprocedural hemostasis time during the follow-up period.

**Results:**

The technical success of distal transradial access without major complications was 97.9% (46/47). Of the 46 patients, one patient (2.2%) had a minor complication, which was a thrombotic segmental occlusion of the distal radial artery. Of the enrolled patients, only one patient did not complete the transradial access procedure via the snuffbox because the left proximal subclavian artery was occluded and a crossover to conventional transfemoral access was performed. The mean postprocedural hemostasis time was 131.7 minutes (range, 120–360 minutes).

**Conclusion:**

Distal transradial access can be a valid option for the endovascular treatment of various noncoronary interventions with technical feasibility and safety.

## Introduction

Conventionally, transfemoral access (TFA) is commonly preferred for diagnostic angiography and endovascular treatment. In particular, transradial access (TRA) has been preferred for coronary angiography because of better patient recovery after the procedure and fewer vascular complications at the puncture site compared to transfemoral or transbrachial access [1–3]. Therefore, interventional procedures with TRA have been selectively used in the peripheral arterial system.

TRA is safe because the hands have a dual supply system via radial and ulnar arteries connecting the palmar arch. Because of these anatomic characteristics, distal perfusion of the hand is rarely affected, even in cases of radial or ulnar artery occlusion. However, despite this advantage, radial artery occlusion remains a major concern [4].

**Fig 1:**
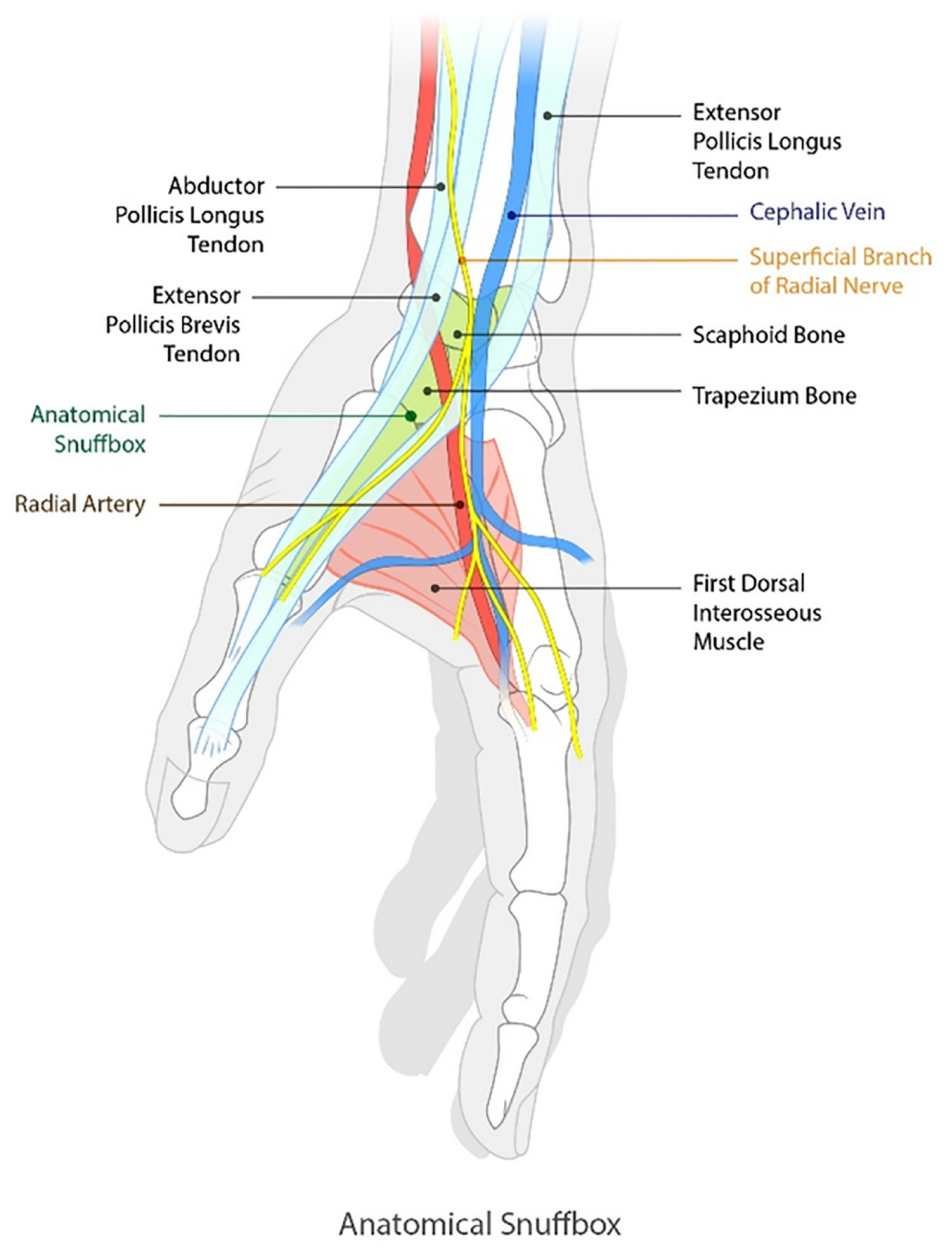
Anatomy of the snuffbox. The snuffbox is bounded by the tendons of the extensor pollicis brevis and the abductor pollicis longus laterally (radial side), and by the tendon of the extensor pollicis longus medially (ulnar side). The inside of the snuffbox consists of the deep palmar branch of the RA and the superficial branch of the radial nerve and cephalic vein (variable).

Recently, distal transradial access (dTRA) in the anatomical snuffbox has been introduced as a new technique for access [5, 6]. Anatomically, the snuffbox is a triangular-shaped depression on the radial dorsal aspect of the hand, and the distal radial artery under the snuffbox, passing along the scaphoid and trapezium, is the deep palmar branch of the radial artery (Fig 1). The deep palmar branch is less relevant to finger ischemia because the superficial branch is the main supply to the digital arteries [6, 7]. Therefore, iatrogenic occlusion of the distal radial artery at the snuffbox seems to be tolerable and the problem of radial artery occlusion seems to have been overcome by dTRA.

In this study, we aimed to evaluate the feasibility and safety of dTRA for both conventional angiography and endovascular treatment of noncoronary interventions in various organs in a multicenter study to validate its use in clinical practice.

## Materials and methods

### Study population

This retrospective analysis was based on patient data collection from two institutions. The Institutional Review Board approved this study and the requirement for informed consent was waived because of the retrospective study design (Ewha Womans University Seoul Hospital Institutional Review Board (IRB 2020-02-028-001) and Gyeongsang National University Changwon Hospital Institutional Review Board (GNUCH 2020-02-026). Between January 2018 and December 2019, 46 patients underwent dTRA for both conventional angiography and endovascular treatment.

### Technical details of the distal TRA

**Fig 2:**
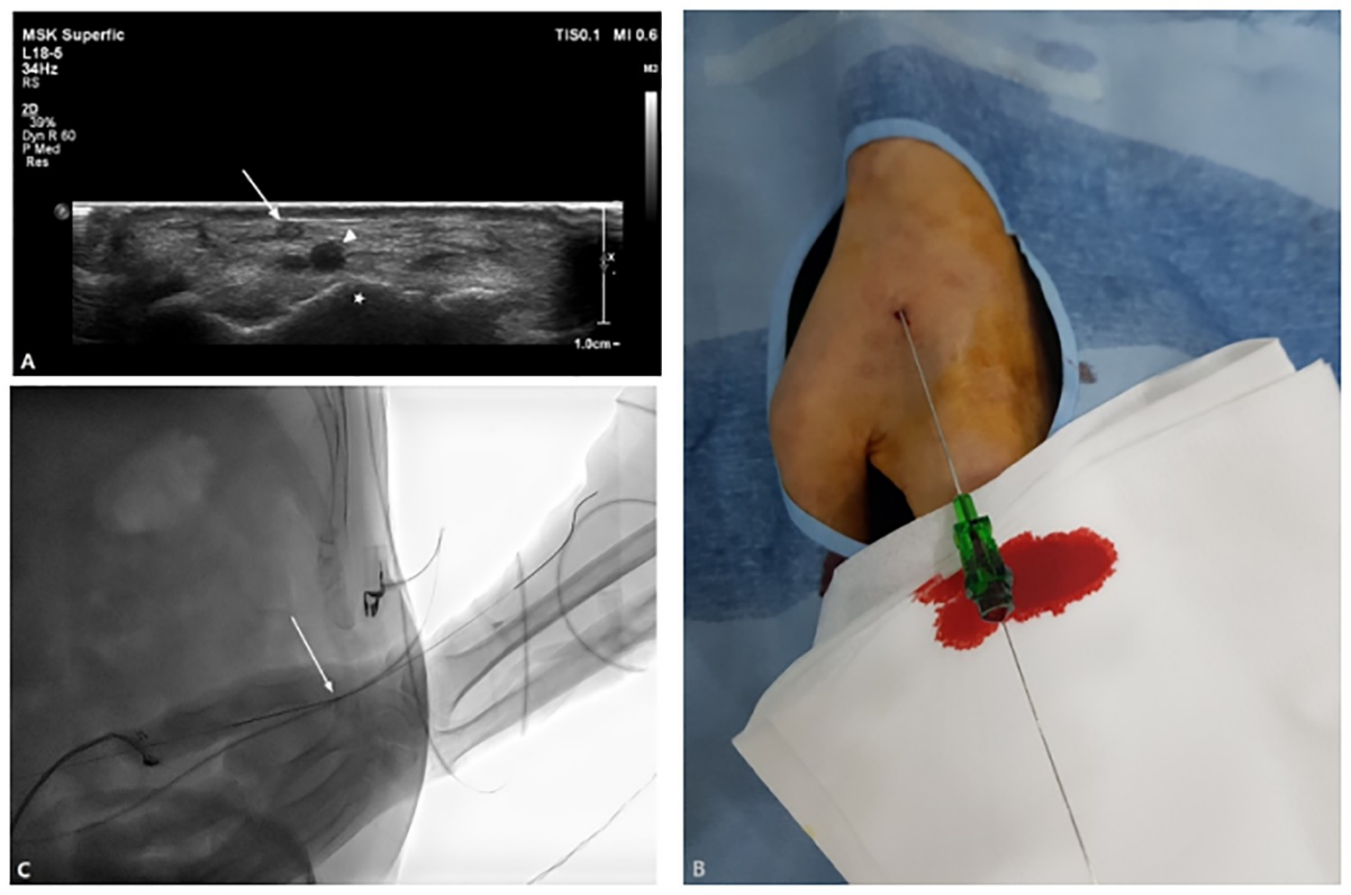
**(a)** The ultrasound image of the anatomical snuffbox shows the superficial branch of the radial nerve (arrow), the distal radial artery (arrowhead), and the trapezium bone (asterisk). **(b)** The distal radial artery at the snuffbox was punctured using a microneedle. **(c)** The fluoroscopic image shows the punctured distal radial artery at the snuffbox (arrow) and the guidewire passed through the proximal radial artery.

**Fig 3:**
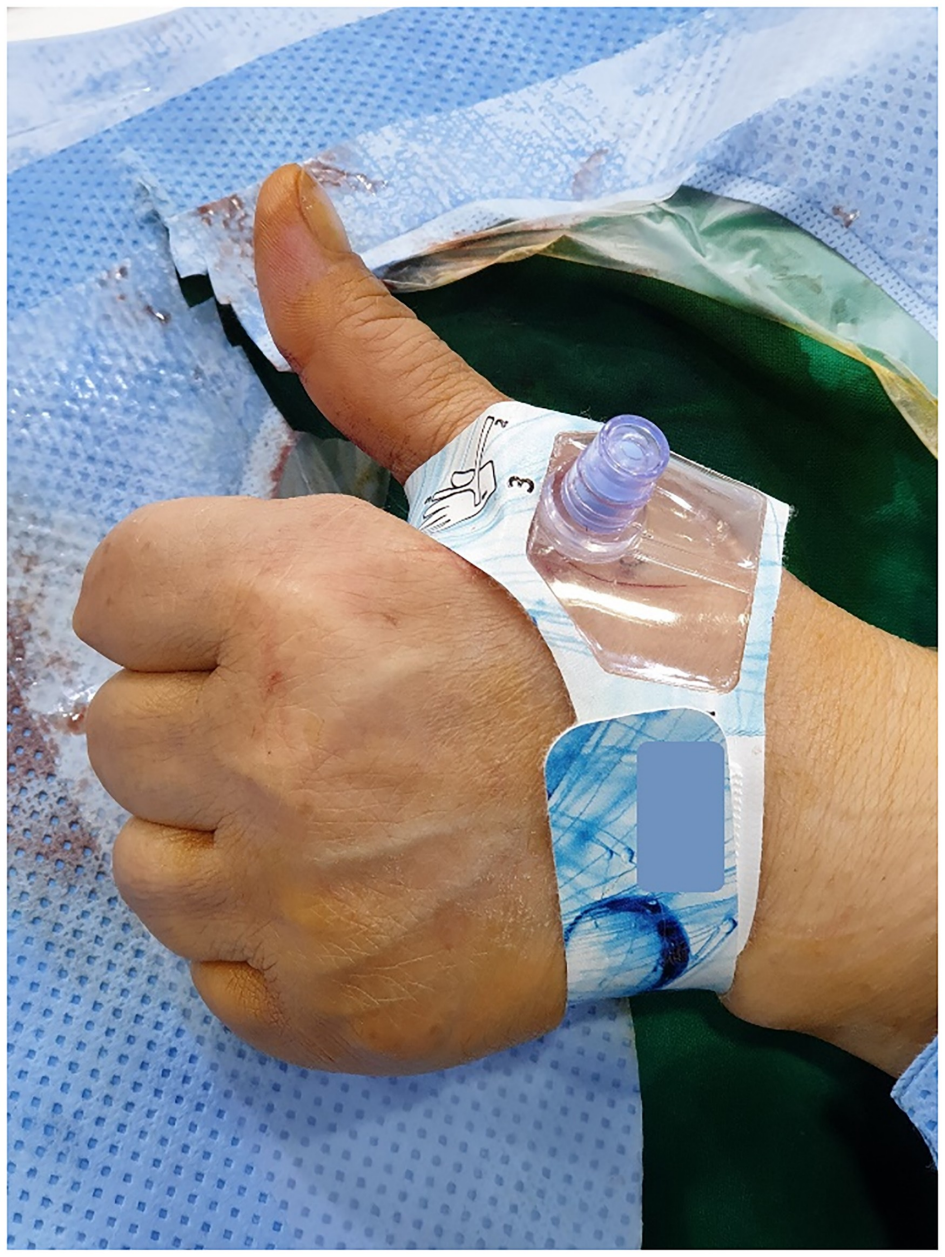
The pneumatic radial compression device applied after the removal of the vascular sheath.

1. Pre-procedural examination. Distal radial artery flow and diameters at the snuffbox were evaluated by ultrasonography (USG) and a Barbeau test was performed in each patient.
2. Distal TRA at the anatomical snuffbox. In the supine position, the patient’s left hand was gently placed near the right groin region in the pronation position. After local anesthesia, the distal radial artery at the snuffbox was accessed under USG guidance using a 5- or 6-Fr transradial kit (Prelude, Merit Medical, South Jordan, UT, USA; or Radiofocus, Terumo, Tokyo, Japan) (Fig 2).
3. Conventional angiography and endovascular procedure. Conventional angiography was performed coaxially with a 0.035-inch hydrophilic guidewire (Terumo, Tokyo, Japan) and a 5-Fr catheter system of suitable length and shape for the procedure. If necessary, a coaxial 1.9- or 2.0-Fr, 130- or 150-mm, microcatheter (Radio Star, Taewoong Medical, Gimpo, South Korea; or Progreat, Terumo, Tokyo, Japan) was used. In case of subclavian artery stenting among endovascular procedures, angioplasty was performed for subclavian artery stenosis using 7mm x 6cm (4Fr system, Pulsar-18 SE nitinol stent, BIOTTRONIK AG, Bülach, Swizerland) or 10mm x 6cm (6Fr system, Epic, Boston Scientific Corp., Natric, MA, USA) self expandible stent.
4. Anticoagulation and vasodilators. After confirming the radial access, 200 μg of nitroglycerin and 2,000 IU of heparin were administered through the vascular sheath. After 90 minutes, an additional 1,000 IU of heparin was instilled every 60 minutes during the procedure. After completion of the treatment, an additional 200 μg of nitroglycerin was administered through the vascular sheath.
5. Hemostasis of the access site. The vascular sheath was removed immediately after completion of the procedure. Subsequently, a pneumatic radial compression device (PreludeSYNC DISTAL, Merit Medical) was used for two hours (Fig 3). After a physical examination to assess hemostasis, the compression device was carefully removed, and complete hemostasis was confirmed with Doppler USG.
6. Follow-up. Complete hemostasis was confirmed for all patients the next day in the angiography suite by both physical examination and Doppler USG. Moreover, distal radial artery patency was confirmed by Doppler USG and other complications were also assessed. Routine follow-up was performed in each patient to evaluate distal radial artery patency at each outpatient office visit by physical examination and Doppler USG.

### Study outcome and definition

Technical success was defined as completion of the planned dTRA procedure without changing the access site. For endovascular treatment, the non-targeted release of embolic agents, insufficient embolization, or misplacement of the stent were considered technical failures. The time required for arterial puncture was recorded. The patency of the distal radial artery at the snuffbox was recorded immediately after the procedure, one day after the procedure, and at follow-up by Doppler USG.

All complications related to access site were recorded during the follow-up period. The classification of the complications was based on the quality improvement guidelines published by the Society of Interventional Radiology (SIR) [8]. Neurological complications were documented according to the National Cancer Institute Common Terminology Criteria for Adverse Events version 5.0 (NCI CTCAE v5.0). Major complications included need for prolonged hospitalization, unplanned increase in the level of care administered, permanent adverse sequelae, and death. Minor complications included need for additional nominal therapy, overnight admission for observation, the loss of radial pulse without evidence of distal ischemia, and hematoma or blood loss not requiring transfusion or open surgical repair [8–11].

## Results

We performed 47 procedures through the dTRA in 46 patients (36 men and 10 women; age range, 37–91 years; mean age, 64 years). The demographic characteristics of the patients are presented in Table 1 and procedural characteristics and selected vessels for angiography are presented in Tables 2 and 3.

**Table 1:** Clinical characteristics of 46 patients.

Characteristic	Value
Age (years), mean (range)	64 (37–91)
Sex	
Male	36 (78.3)
Female	10 (21.7)
Hypertension	14 (30.4)
Diabetics	13 (28.3)
Smoking	16 (34.8)

The data presented in parentheses are the percentages of each item.

**Table 2:** Types of procedures performed.

Characteristic	Value
Intervention	
TACE	19 (40.4)
Bronchial artery embolization	7 (14.9)
Renal artery intervention	3 (6.4)
Angioplasty of dysfunctional AVF	7 (14.9)
Subclavian artery stenting	5 (10.6)
Uterine artery embolization	1 (2.1)
Other embolization	5 (10.6)
Barbeau test response	
A	21 (44.7)
B	20 (42.6)
C	6 (12.7)
D	0 (0)
Crossover to TFA	1 (2.1)
Sheath size	
5-French	37 (78.7)
6-French	10 (21.3)

The data presented in parentheses are the percentage of each item. AVF, arteriovenous fistula; TACE, transcatheter arterial chemoembolization; TFA, transfemoral access.

**Table 3:** Selected vessels for angiography and the catheters used.

Selected vessel	Applied catheter	Number
Celiac trunk	Ultimate 1*, Davis	22
Renal artery	Davis, H1*	3
Subclavian artery	Davis*, H1	5
Internal mammary artery	Davis*, Pigtail	4
Intercostal artery	Davis, Headhunter*	5
Bronchial artery	Davis, Headhunter*	7
Lateral thoracic	Davis	3
Superior mesenteric artery	Ultimate 1	3
Internal iliac artery	Davis	1
Radial artery	KMP	7

Davis, 5-Fr x 125 cm Davis catheter (Jung Sung Medical, Seongnam, South Korea); Headhunter, 5-Fr x 100 cm Headhunter catheter (Jung Sung Medical, Seongnam, South Korea); H1, 5-Fr x 110 cm H1 Torcon Advantage catheter (Cook Medical, Bjaeverskov, Denmark); KMP, 5-Fr x 40 cm KMP catheter (Cook Medical, Indiana, USA); Pigtail, 5-Fr x 80 cm straight pigtail catheter (Terumo Outlook, Tokyo, Japan); Ultimate 1, 5-Fr x 125 cm Ultimate 1 Performa catheter (Merit Medical, South Jordan, UT, USA).

* routine choice.

The following endovascular procedures were performed: chemoembolization for hepatocellular carcinoma (19/47, 40.4%), bronchial artery embolization for hemoptysis (7/47, 14.9%), renal intervention (3/47, 6.4%) [embolization for renal angiomyolipoma (1/47, 2.1%), embolization of renal artery aneurysm (1/47, 2.1%), embolization of the renal artery for hematuria after renal biopsy (1/47, 2.1%)], angioplasty for vascular access for hemodialysis (7/47, 14.9%), angioplasty with stenting for subclavian artery stenosis (5/47, 10.6%), uterine artery embolization for post-partum bleeding (1/47, 2.1%), embolization for other causes (5/47, 10.6%) [embolization for gastric cancer bleeding (2/47, 4.3%), embolization for metastatic hepatic tumor bleeding (1/47, 2.1%), embolization for empyema necessitatis (1/47, 2.1%), and embolization for lung cancer bleeding (1/47, 2.1%)].

Technical success was achieved in 46 procedures (97.9%). In one patient (2.2%), successful arterial access could be performed with dTRA; however, the procedure could not be completed because of an unexpected left subclavian artery occlusion. The planned procedure was successfully completed with a crossover to the access site using TFA.

The required time for arterial access was 3.9 ± 1.1 min (mean ± standard deviation [SD]; range, 3.0–7.0 min) and was 131.7 ± 41.2 min (mean ± SD; range 120–360 min) for hemostatic compression. No major procedural complications were noted. The vascular sheath at the access site was immediately removed after completion of the procedure in all 46 patients and pneumatic compression devices were used. Successful hemostasis was confirmed by physical examination and Doppler USG on the day of the procedure. On the following day, radial artery patency without complications was confirmed by Doppler USG in 45 of the 46 patients. Through Doppler USG, one patient had demonstrated segmental thrombotic occlusion of the distal radial artery at the puncture site, which was asymptomatic and did not present neurological complication. The distal arterial flow patency in this patient was observed on Doppler USG and no additional treatment was performed for the occlusion. At 3-month follow-up Doppler USG, the patient had demonstrated spontaneous recanalization of the distal radial artery. No other complications were recorded during the follow-up period.

The follow-up period was 408.7 ± 203.9 days (mean ± SD; range 30–686 days). One patient died during the follow-up period. The initial distal radial artery diameter of 46 patients was 2.3 ± 0.2 mm (mean ± SD; range, 2.0–2.9 mm). The change in diameter of the distal radial artery during follow-up was 0.09 ± 0.1 mm (mean ± SD; range, 0–0.5 mm). The distal radial artery diameter of the patient with segmental thrombotic occlusion decreased the most.

## Discussion

In the current study, we assessed the technical feasibility of dTRA in the endovascular treatment of various noncoronary interventions. The dTRA procedure demonstrated a high technical success rate and low procedural complication rate that were comparable with conventional TRA or TFA. In addition, the mean time to achieve dTRA was also comparable to that of conventional TRA [12].

Endovascular treatment is the preferred procedure for treating vascular diseases such as dysfunctional arteriovenous fistula and steno-occlusive subclavian artery disease, and bleeding or tumor embolization [13–18]. The technical success or safety of the endovascular procedure has been sufficiently discussed in various fields [19–21]. Therefore, further interest has focused on the convenience of patients undergoing procedure rather than on its performance.

Although TFA is associated with disadvantages such as long-term immobilization of patients due to hemostasis and vascular complications (e.g. bleeding, hematoma, pseudoaneurysm, arteriovenous fistula, and thrombosis), it has been commonly used for conventional angiography and endovascular treatment. The incidence of these complications ranges from 0% to 17% [22–26]. To avoid or minimize these complications, TRA was introduced in 1989 as an alternative access technique for coronary angiography [27]. Several studies have verified the safety and efficacy of TRA, and more recently, TRA was recommended as the primary access technique for coronary endovascular procedures [3, 9, 28–30].

Despite previous studies supporting the TRA approach, some subsequent studies revealed the risk of access site complications such as radial arterial occlusion [4, 9]. Therefore, a new access technique, dTRA, was developed for coronary angiography and neurointerventional procedures [5–7, 31]. An advantage of dTRA is that it focuses on a collateral system, the palmar arch, to ensure sustained blood flow in light of unpredictable interruptions.

However, the majority of reports on the use of dTRA have focused on coronary interventions and neurointerventional procedures. There has been only one previous study on the dTRA approach for noncoronary interventions; however, the study did not explore the use of dTRA in various procedures because majority of the study’s patients underwent hepatocellular carcinoma-related procedures [32].

In the present study, dTRA was used for various endovascular treatments such as chemoembolization, embolization, angioplasty, stenting of various organs, and diagnostic angiography with a high rate of technical success. Only one case of asymptomatic segmental occlusion of the distal radial artery was reported. These results are supported by the fact that the dTRA approach utilizes the advantages of the TRA approach and complements its disadvantages. Moreover, unlike the operator’s position during TRA, the operator’s experience with dTRA is similar to that during TFA, where the left hand is placed next to the right groin. This is another advantage of dTRA over conventional TRA.

In this study, the time required for arterial access using dTRA was not significantly different from the time previously reported for conventional TRA [12], even though there might be differences depending on the degree of operator experience in clinical practice. There were some groups that carried out the puncture without ultrasound guidance [33], but we conducted USG-guided puncture for safety because the distal radial artery was small in diameter and short in length of the snuffbox segment. In addition, the administration of anticoagulants and vasodilators after vascular sheath insertion prevents spasm, thrombosis, or occlusion of the small distal radial artery at the snuffbox. We found little changes in the diameter of the distal radial artery at the snuffbox in most patients during the follow-up period (mean, 408.7 days).

This study had some limitations. First, there was limited availability of catheters, and these catheters differed in shape and length. Second, the operators increased exposure to radiation, particularly during the steepest part of the learning curve, was a drawback to the study [34]. Third, the present study design was retrospective and did not have a comparative control cohort. Fourth, the study included a limited number of cases. Lastly, only the Barbeau test was used to categorize patients as controls; however, the Barbeau test alone cannot determine the presence of proximal artery stenosis or occlusion. Proximal upper extremity artery evaluation by USG or by using the ankle-brachial pressure index should be considered before performing a dTRA procedure.

In conclusion, this was the first validation study to assess the efficacy of dTRA for performing noncoronary interventions in multiple organs. DTRA can be a well-suited, safe, and feasible access option in the field of noncoronary interventions.

## Supporting information

S1 File (XLSX)
